# Full-Stokes polarization multispectral images of various stereoscopic objects

**DOI:** 10.1038/s41597-023-02184-1

**Published:** 2023-05-27

**Authors:** Axin Fan, Tingfa Xu, Geer Teng, Xi Wang, Yuhan Zhang, Chang Xu, Xin Xu, Jianan Li

**Affiliations:** 1 Key Laboratory of Photoelectronic Imaging Technology and System of Ministry of Education of China, School of Optics and Photonics, Beijing Institute of Technology, Beijing, 100081 China; 2 Beijing Institute of Technology Chongqing Innovation Center, Chongqing, 401151 China; 3 Department of Engineering Science, Institute of Biomedical Engineering, University of Oxford, Oxford, OX3 7DQ UK; 4 School of Printing & Packaging Engineering, Beijing Institute of Graphic Communication, Beijing, 102600 China

**Keywords:** Imaging and sensing, Optical sensors

## Abstract

Polarization multispectral imaging (PMI) has been applied widely with the ability of characterizing physicochemical properties of objects. However, traditional PMI relies on scanning each domain, which is time-consuming and occupies vast storage resources. Therefore, it is imperative to develop advanced PMI methods to facilitate real-time and cost-effective applications. In addition, PMI development is inseparable from preliminary simulations based on full-Stokes polarization multispectral images (FSPMI). Whereas, FSPMI measurements are always necessary due to the lack of relevant databases, which is extremely complex and severely limits PMI development. In this paper, we therefore publicize abundant FSPMI with 512 × 512 spatial pixels measured by an established system for 67 stereoscopic objects. In the system, a quarter-wave plate and a linear polarizer are rotated to modulate polarization information, while bandpass filters are switched to modulate spectral information. The required FSPMI are finally calculated from designed 5 polarization modulation and 18 spectral modulation. The publicly available FSPMI database may have the potential to greatly promote PMI development and application.

## Background & Summary

Polarization multispectral imaging (PMI) acquires four-dimensional information in polarization, spectral and spatial domains. Benefiting from the rich information acquired, PMI has been attracting widespread attention from researchers in various fields. Concretely, PMI shows great significance for target detection involving specular reflection inpainting^1^, background segmentation^2^ and tensor representation^3^. In remote sensing, PMI is closely related to marsh vegetation classification^4^, coastal wetland classification^5^ and leaf nitrogen determination^6^. PMI also develops rapidly in biological diagnosis, demonstrating compelling effectiveness in revealing skin complications of diabetes mellitus^7^ and characterizing microstructures of cancerous tissues^8^. Obviously, the stability, reliability, efficiency and universality of imaging methods determine the popularity of PMI technology in various fields.

Generally, PMI is being improved in both imaging systems and reconstruction algorithms. Snapshot channeled imaging spectropolarimeter usually consists of multiple retarders and polarizers, and then reconstructs information based on Fourier transform^9^ or neural networks^10^. Snapshot division imaging spectropolarimeter captures multiple images simultaneously by adopting a four-quadrant retarder array^11^, a staircase-grid lenslet array^12^ or a pixelated micro-polarization array^13^. There is no doubt that optical device division requires advanced technology and prohibitive cost. Furthermore, compressive sensing (CS) theory^14^ enables conventional optical devices for compressive imaging spectropolarimeter, such as retarder^15–19^, polarizer^15,17^, coded aperture^15^, double Amici prism^15^, Wollaston prism^16^, digital micromirror devices^18^ and liquid crystal tunable filter (LCTF)^17–19^. Recently, significant breakthroughs in PMI are often associated with novel materials. Full-Stokes polarization camera can be implemented by designing diffraction grating metasurfaces^20^, pixelated polarizer metasurfaces^21^, or vectorial Fourier metasurfaces^22^. In addition, spectral and polarization information can be obtained simultaneously by exploiting organic photodetectors^23,24^. These novel materials greatly simplify imaging systems without standard optics.

Nonetheless, all PMI improvements require simulation validation based on full-Stokes polarization multispectral images (FSPMI). Obviously, it can be found that FSPMI adopted for validation are varied in current studies due to the lack of relevant databases. This phenomenon not only increases the costs and difficulties of measuring FSPMI experimentally, but also prevents researchers from efficiently comparing existing imaging methods based on uniform FSPMI. On the contrary, other publicly available databases^25–27^ are extremely conducive to the multidimensional and extensive development of related technologies.

In this paper, we therefore publicize an FSPMI database, including four Stokes parameters, 18 spectral bands, 512 × 512 spatial pixels and 67 stereoscopic objects. The FSPMI database is carefully measured by establishing an experimental system consisting mainly of a quarter-wave plate (QWP), a linear polarizer (LP), 18 bandpass filters and a complementary metal oxide semiconductor (CMOS) detector. By switching the bandpass filters, the broadband spectral intensities of the object reflected light are modulated and compressed into multiple narrowband spectral intensities to be sequentially detected by the CMOS. Meanwhile, by rotating the fast axis of QWP and the transmission axis of LP, the four Stokes parameters of the object reflected light are modulated and compressed into a new Stokes parameter representing the total light intensity to be also detected by the CMOS. The four Stokes parameters of the object reflected light are then calculated from the light intensities detected under five polarization modulation. Various stereoscopic objects are selected for database measurement because the polarization information can reflect the surface texture of objects. The FSPMI database is likely to enhance PMI technology development in terms of simulation validation and application popularity.

## Methods

### Experimental system establishment

**Fig. 1:**
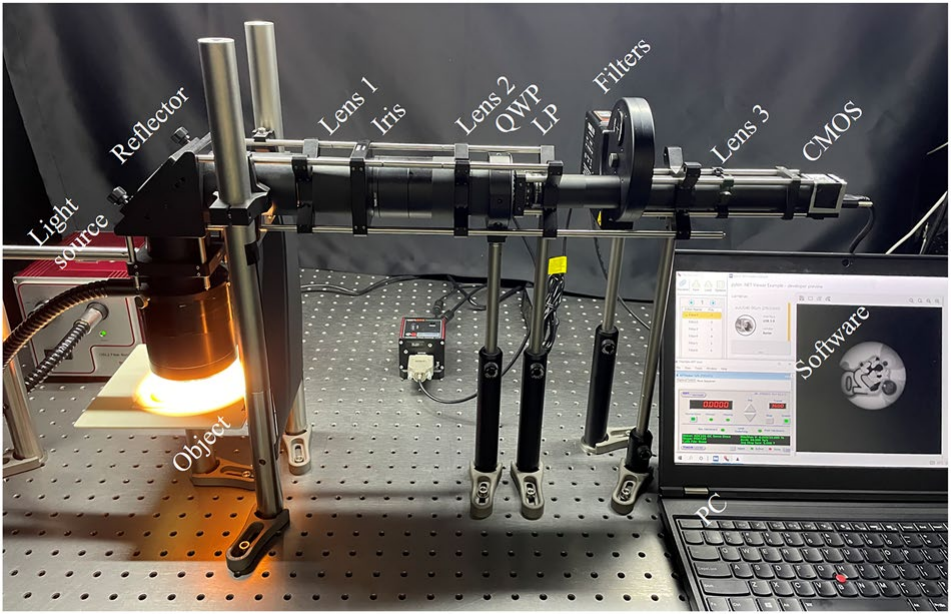
The established experimental system for measuring full-Stokes polarization multispectral images (FSPMI).

To measure FSPMI, we first establish an experimental system as shown in Fig. 1. The light emitted from a light source is initially reflected by an object, then passed through a reflector, lens 1, an iris, lens 2, a QWP, an LP, 18 bandpass filters, lens 3 and finally detected by a CMOS. Both lens 1 and lens 3 are imaging lenses, while lens 2 is a collimating lens. The iris is a field stop to prevent overlapping of images. The entire cage system is established in a dark room and connected by several lens tubes to further block stray light. The manufacturers, items and key parameters of each optical component established in the experimental system are clearly listed in Table 1. Obviously, the operating wavelength range (OWR) of the system is restricted by the 18 bandpass filters, and the optical propagation size is limited by the QWP with the minimum diameter of 12.7 mm. Ignoring the fluctuation of only 2 nm, the central wavelength (CWL) of the 18 bandpass filters ranges from 520 nm to 690 nm at 10 nm intervals, and the full width at half maximum (FWHM) is about 10 nm. During the establishment of the system, the object platform is fixed within the optimal working distance range of 50 mm to 140 mm from the ring illuminator. The positions of the three lenses are then carefully adjusted according to their focal length to obtain the clearest object image on the CMOS. To facilitate experimental data measurement, the QWP is mounted in a motorized precision rotation stage, the LP is mounted in a cage rotation mount, and each of the 6 adjacent bandpass filters is mounted in a 6-position filter wheel.

**Table 1:** The manufacturers, items and key parameters of each optical component established in the experimental system.

Component name	Manufacturer	Item #	Key parameter
Light source	Thorlabs	OSL2	OWR = 400–1600 nm
Ring illuminator	Thorlabs	FRI61F50	OWR = 365–900 nm, Optimal working distance = 50–140 mm
Reflector	Thorlabs	BB2-E02	Ø50.8 mm, OWR = 400–750 nm
Reflector mount	Thorlabs	KCB2/M	60 mm right-angle mount, 45° installation, ± 4° adjustment
Lens 1	Thorlabs	AC254-040-A	Ø25.4 mm, f = 40 mm, OWR = 400–700 nm
Iris	Thorlabs	SM2D25	Ø1–25 mm
Lens 2	Thorlabs	AC254-125-A	Ø25.4 mm, f = 125 mm, OWR = 400–700 nm
QWP	Thorlabs	SAQWP05M-700	Ø12.7 mm, 25.4 mm case, OWR = 325–1100 nm
QWP stage	Thorlabs	KPRM1E/M	Ø25.4 mm, 360° motorized rotation
LP	Thorlabs	LPVISC100-MP2	Ø25.0 mm, OWR = 510–800 nm
LP mount	Thorlabs	CRM1/M	Ø25.4 mm, 360° continuous rotation
Filter 1	Thorlabs	FB520–10	Ø25.4 mm, CWL = 520 ± 2 nm, FWHM = 10 ± 2 nm
Filter 2	Thorlabs	FB530–10	Ø25.4 mm, CWL = 530 ± 2 nm, FWHM = 10 ± 2 nm
Filter 3	Thorlabs	FB540-10	Ø25.4 mm, CWL = 540 ± 2 nm, FWHM = 10 ± 2 nm
Filter 4	Thorlabs	FB550-10	Ø25.4 mm, CWL = 550 ± 2 nm, FWHM = 10 ± 2 nm
Filter 5	Thorlabs	FB560-10	Ø25.4 mm, CWL = 560 ± 2 nm, FWHM = 10 ± 2 nm
Filter 6	Thorlabs	FB570-10	Ø25.4 mm, CWL = 570 ± 2 nm, FWHM = 10 ± 2 nm
Filter 7	Thorlabs	FB580-10	Ø25.4 mm, CWL = 580 ± 2 nm, FWHM = 10 ± 2 nm
Filter 8	Thorlabs	FB590-10	Ø25.4 mm, CWL = 590 ± 2 nm, FWHM = 10 ± 2 nm
Filter 9	Thorlabs	FB600-10	Ø25.4 mm, CWL = 600 ± 2 nm, FWHM = 10 ± 2 nm
Filter 10	Thorlabs	FB610-10	Ø25.4 mm, CWL = 610 ± 2 nm, FWHM = 10 ± 2 nm
Filter 11	Thorlabs	FB620-10	Ø25.4 mm, CWL = 620 ± 2 nm, FWHM = 10 ± 2 nm
Filter 12	Thorlabs	FB630-10	Ø25.4 mm, CWL = 630 ± 2 nm, FWHM = 10 ± 2 nm
Filter 13	Thorlabs	FB640-10	Ø25.4 mm, CWL = 640 ± 2 nm, FWHM = 10 ± 2 nm
Filter 14	Thorlabs	FB650-10	Ø25.4 mm, CWL = 650 ± 2 nm, FWHM = 10 ± 2 nm
Filter 15	Thorlabs	FB660-10	Ø25.4 mm, CWL = 660 ± 2 nm, FWHM = 10 ± 2 nm
Filter 16	Thorlabs	FB670-10	Ø25.4 mm, CWL = 670 ± 2 nm, FWHM = 10 ± 2 nm
Filter 17	Thorlabs	FB680-10	Ø25.4 mm, CWL = 680 ± 2 nm, FWHM = 10 ± 2 nm
Filter 18	Thorlabs	FB690-10	Ø25.4 mm, CWL = 690 ± 2 nm, FWHM = 10 ± 2 nm
Motorized wheel	Thorlabs	FW102C	Ø25 mm, 6-position filter, ± 2° accuracy
Replaceable wheel	Thorlabs	FW102CW	Ø25 mm, 6-position filter
Lens 3	Thorlabs	AC254-075-A	Ø25.4 mm, f = 75 mm, OWR = 400–700 nm
CMOS	Basler	acA2040-180km	1024 × 1024 pixels, 11.0 μm pixel pitch

(OWR: operating wavelength range, CWL: central wavelength, FWHM: full width at half maximum).

### Image measurement

#### System adjustment

**Fig. 2:**
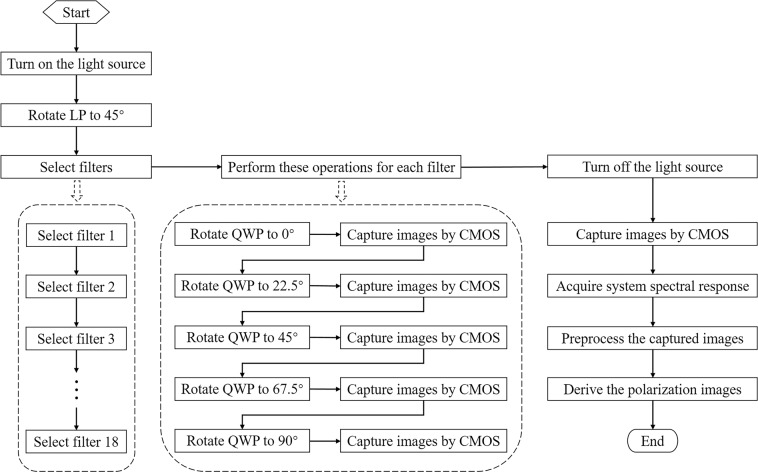
The specific experimental procedure for measuring full-Stokes polarization multispectral images (FSPMI). In the measurement, the step “Capture images by CMOS” is repeated 6 times.

Figure 2 illustrates the specific experimental procedure for measuring FSPMI. Firstly, the light source is turned on and preheated for about 10 min. During this period, the object placed on the fixed platform is moved and rotated slowly in the horizontal plane to optimize the object image in the field of view of CMOS. Thus, the object region of interest is imaged within the specified 512 × 512 pixel region near the CMOS center. Meanwhile, based on the pixel format of Mono8 and the fixed exposure time of 10000 μs on CMOS, the intensity of the light source is adjusted carefully so that the maximum value of the images captured under 18 bandpass filters is approximately 230. The light source adjustment is to ensure that the captured images will not be overexposed throughout the experiment. In other words, the object position and the intensity of the light source are fixed by the above operations.

### Image measurement

#### Image capture

Then, the transmission axis of LP is rotated to 45° and fixed thereafter. Select a bandpass filter to perform the following polarization measurements. That is, rotate the fast axis of QWP to 0° to capture 6 images consecutively by CMOS for further averaging, and then rotate the fast axis of QWP to 22.5°, 45°, 67.5° and 90° to similarly capture the images respectively. Notably, the 18 bandpass filters are mounted in three 6-position filter wheels. By carefully replacing the filter wheels manually, the 18 bandpass filters are switched to perform the polarization measurements separately to capture a total of 540 images. Finally, the light source is turned off and 6 images are continuously captured by CMOS to reflect the dark noise of the detector. As described above, the entire process of image acquisition for an object takes about 35 min. Generally, the above steps are repeated several hours later to capture images for the next object. In total, the required images are captured for 67 plastic objects in about 19 days. The third object is 3D-printed and glued, while the remaining 66 objects are purchased refrigerator magnets.

### Image preprocessing

#### System fluctuation and dark noise elimination

The captured images are preprocessed separately for each object to minimize the inherent influence of the experimental system. The image preprocessing is divided into four steps: system fluctuation reduction, dark noise elimination, spectral response calibration, image maximum normalization. In order to reduce the influence of system fluctuation, the 6 images captured continuously under the same bandpass filter and QWP angle are averaged to obtain a total of 90 images for each object. The 6 images captured with the light source off are also averaged to obtain dark noise for each experiment. The averaged dark image is further subtracted from each of the averaged object images to eliminate the influence of dark noise. Moreover, the pixel value less than zero in the object image with dark noise eliminated is assigned as zero to satisfy the rationality of the captured image.

#### Spectral response acquisition

**Fig. 3:**
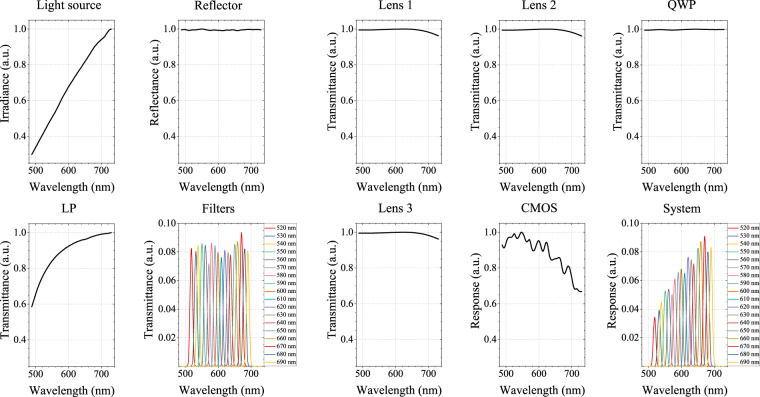
The normalized spectral characteristics of adopted optical devices and the entire system according to the manufacturer specifications.

**Fig. 4:**
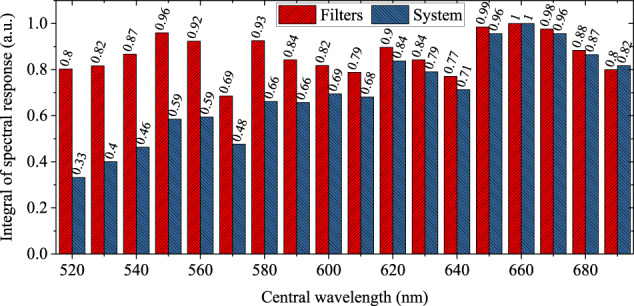
The integral value of spectral response after normalization of 18 bandpass filters and the entire system.

In addition, the spectral response of the system is determined by the combination of adopted optical devices. Figure 3 successively shows the normalized spectral characteristics of the light source, reflector, lens 1, lens 2, QWP, LP, 18 bandpass filters, lens 3, CMOS detector and the entire system. Based on standard measurements provided by the manufacturer, the spectral characteristics of each optical device are obtained by linear interpolation to 196 wavelengths ranging from 488.41 nm to 730.12 nm and then smoothing. The spectral characteristics of all optical devices are multiplied at each wavelength to obtain the spectral response of the entire system. Meanwhile, the spectral maximum is normalized for each optical device. In particular, the spectral response of each spectral band at all 196 wavelengths is integrated by the trapezoidal method. The 18 bandpass filters and the entire system are then normalized to the maximum integral value across all spectral bands, respectively. The integral value of spectral response after normalization of 18 bandpass filters and the entire system is shown in Fig. 4.

#### Spectral response calibration and image normalization

**Fig. 5:**
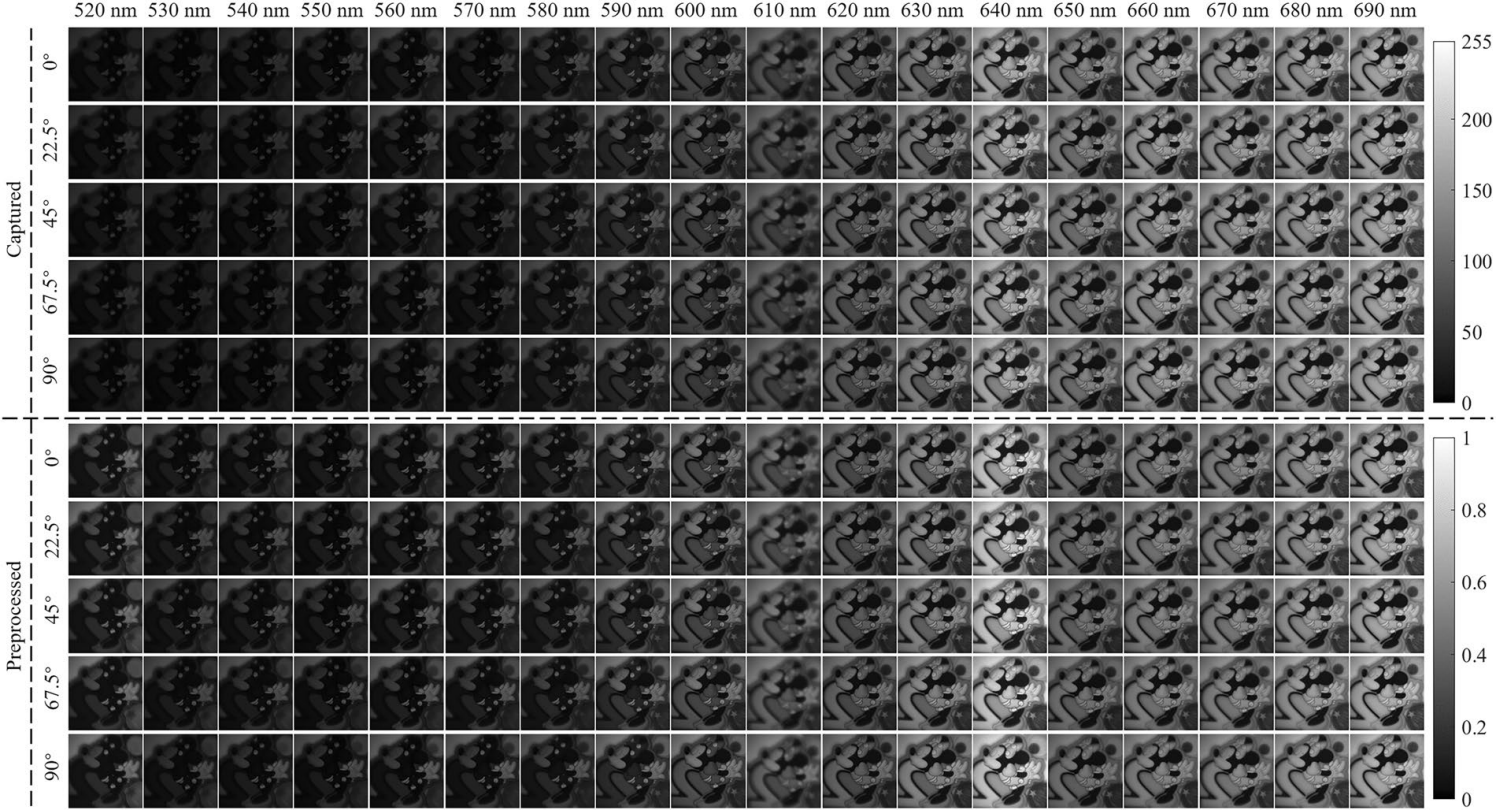
The images captured and preprocessed for an object at 18 spectral bands and five QWP angles.

The spectral response calibration is then performed for the object image that eliminates the system fluctuation and dark noise. Specifically, the object image under each spectral band is divided by the integral value of the spectral response of the entire system under the corresponding spectral band. Finally, image normalization is carried out for each object based on the maximum value of all 90 images. Figure 5 comprehensively shows the images captured and preprocessed for an object at 18 spectral bands and five QWP angles. The captured images are displayed well in the grayscale range of 0 to 255, while the preprocessed images are displayed well in the grayscale range of 0 to 1. Obviously, image preprocessing can significantly enhance image brightness at short spectral bands to further compensate for the brightness difference caused by the uneven spectral response of the system. Moreover, image preprocessing can be further improved by experimentally measuring the spectral response of the system instead of the standard measurements provided by the manufacturer.

### Full-Stokes polarization modeling

#### Polarization modulation theory

Full-Stokes images are calculated from the preprocessed images according to the designed polarization modulation strategy. In the experimental system, QWP and LP jointly modulate the polarization information of the light reflected by the object. Let *S*_0_, *S*_1_, *S*_2_, *S*_3_ represent the four Stokes parameters of the object light. Assuming that the fast axis of QWP is rotated to an angle *θ*(0° ≤ *θ<*180°), then the Mueller matrix of QWP can be expressed as follows:

1 $${{\bf{M}}}_{1}=\left[\begin{array}{cccc}1 & 0 & 0 & 0\\ 0 & {{\rm{\cos }}}^{2}\left(2\theta \right) & {\rm{\cos }}\left(2\theta \right){\rm{\sin }}\left(2\theta \right) & -{\rm{\sin }}\left(2\theta \right)\\ 0 & {\rm{\cos }}\left(2\theta \right){\rm{\sin }}\left(2\theta \right) & {{\rm{\sin }}}^{2}\left(2\theta \right) & {\rm{\cos }}\left(2\theta \right)\\ 0 & {\rm{\sin }}\left(2\theta \right) & -{\rm{\cos }}\left(2\theta \right) & 0\end{array}\right].$$

Thus, the four Stokes parameters modulated by QWP, denoted as ${S}_{0}^{1}$, ${S}_{1}^{1}$, ${S}_{2}^{1}$, ${S}_{3}^{1}$, can be calculated from

2 $$\left[\begin{array}{c}{S}_{0}^{1}\\ {S}_{1}^{1}\\ {S}_{2}^{1}\\ {S}_{3}^{1}\end{array}\right]={{\bf{M}}}_{1}\times \left[\begin{array}{c}{S}_{0}\\ {S}_{1}\\ {S}_{2}\\ {S}_{3}\end{array}\right]=\left[\begin{array}{cccc}1 & 0 & 0 & 0\\ 0 & {{\rm{\cos }}}^{2}\left(2\theta \right) & {\rm{\cos }}\left(2\theta \right){\rm{\sin }}\left(2\theta \right) & -{\rm{\sin }}\left(2\theta \right)\\ 0 & {\rm{\cos }}\left(2\theta \right){\rm{\sin }}\left(2\theta \right) & {{\rm{\sin }}}^{2}\left(2\theta \right) & {\rm{\cos }}\left(2\theta \right)\\ 0 & {\rm{\sin }}\left(2\theta \right) & -{\rm{\cos }}\left(2\theta \right) & 0\end{array}\right]\times \left[\begin{array}{c}{S}_{0}\\ {S}_{1}\\ {S}_{2}\\ {S}_{3}\end{array}\right].$$

When the transmission axis of LP is rotated to an angle *α*(0° ≤ *α<*180°), the Mueller matrix of LP is

3 $${{\bf{M}}}_{2}=\frac{1}{2}\left[\begin{array}{cccc}1 & {\rm{\cos }}\left(2\alpha \right) & {\rm{\sin }}\left(2\alpha \right) & 0\\ {\rm{\cos }}\left(2\alpha \right) & {{\rm{\cos }}}^{2}\left(2\alpha \right) & {\rm{\cos }}\left(2\alpha \right){\rm{\sin }}\left(2\alpha \right) & 0\\ {\rm{\sin }}\left(2\alpha \right) & {\rm{\cos }}\left(2\alpha \right){\rm{\sin }}\left(2\alpha \right) & {{\rm{\sin }}}^{2}\left(2\alpha \right) & 0\\ 0 & 0 & 0 & 0\end{array}\right].$$

The four Stokes parameters further modulated by LP are denoted as ${S}_{0}^{2}$, ${S}_{1}^{2}$, ${S}_{2}^{2}$, ${S}_{3}^{2}$, which can be similarly calculated by

4 $$\begin{array}{c}[\begin{array}{c}{S}_{0}^{2}\\ {S}_{1}^{2}\\ {S}_{2}^{2}\\ {S}_{3}^{2}\end{array}]={{\bf{M}}}_{2}\times [\begin{array}{c}{S}_{0}^{1}\\ {S}_{1}^{1}\\ {S}_{2}^{1}\\ {S}_{3}^{1}\end{array}]={{\bf{M}}}_{2}\times {{\bf{M}}}_{1}\times [\begin{array}{c}{S}_{0}\\ {S}_{1}\\ {S}_{2}\\ {S}_{3}\end{array}]\\ =\frac{1}{2}[\begin{array}{cccc}1 & \cos (2\alpha ) & \sin (2\alpha ) & 0\\ \cos (2\alpha ) & {\cos }^{2}(2\alpha ) & \cos (2\alpha )\sin (2\alpha ) & 0\\ \sin (2\alpha ) & \cos (2\alpha )\sin (2\alpha ) & {\sin }^{2}(2\alpha ) & 0\\ 0 & 0 & 0 & 0\end{array}]\times [\begin{array}{cccc}1 & 0 & 0 & 0\\ 0 & {\cos }^{2}(2\theta ) & \cos (2\theta )\sin (2\theta ) & -\sin (2\theta )\\ 0 & \cos (2\theta )\sin (2\theta ) & {\sin }^{2}(2\theta ) & \cos (2\theta )\\ 0 & \sin (2\theta ) & -\cos (2\theta ) & 0\end{array}]\times [\begin{array}{c}{S}_{0}\\ {S}_{1}\\ {S}_{2}\\ {S}_{3}\end{array}]\\ =\frac{1}{2}[\begin{array}{cccc}1 & \cos (2\alpha ){\cos }^{2}(2\theta )+\sin (2\alpha )\cos (2\theta )\sin (2\theta ) & \cos (2\alpha )\cos (2\theta )\sin (2\theta )+\sin (2\alpha ){\sin }^{2}(2\theta ) & -\cos (2\alpha )\sin (2\theta )+\sin (2\alpha )\cos (2\theta )\\ \cos (2\alpha ) & {\cos }^{2}(2\alpha ){\cos }^{2}(2\theta )+\cos (2\alpha )\sin (2\alpha )\cos (2\theta )\sin (2\theta ) & {\cos }^{2}(2\alpha )\cos (2\theta )\sin (2\theta )+\cos (2\alpha )\sin (2\alpha ){\sin }^{2}(2\theta ) & -{\cos }^{2}(2\alpha )\sin (2\theta )+\cos (2\alpha )\sin (2\alpha )\cos (2\theta )\\ \sin (2\alpha ) & \cos (2\alpha )\sin (2\alpha ){\cos }^{2}(2\theta )+{\sin }^{2}(2\alpha )\cos (2\theta )\sin (2\theta ) & \cos (2\alpha )\sin (2\alpha )\cos (2\theta )\sin (2\theta )+{\sin }^{2}(2\alpha ){\sin }^{2}(2\theta ) & -\cos (2\alpha )\sin (2\alpha )\sin (2\theta )+{\sin }^{2}(2\alpha )\cos (2\theta )\\ 0 & 0 & 0 & 0\end{array}]\times [\begin{array}{c}{S}_{0}\\ {S}_{1}\\ {S}_{2}\\ {S}_{3}\end{array}].\end{array}$$

#### Full-Stokes derivation

During image capture, CMOS only detects the total light intensity, which is represented by the modulated first Stokes parameter ${S}_{0}^{2}$. Let *I*_*θ,α*_ represent the light intensity information detected by CMOS when the fast axis of QWP and the transmission axis of LP are rotated to angles *θ* and *α* respectively. Therefore, the relationship between the detected information *I*_*θ,α*_ and the four Stokes parameters *S*_0_, *S*_1_, *S*_2_, *S*_3_ of the object light can be written as

5 $${I}_{\theta ,\alpha }={S}_{0}^{2}=\frac{1}{2}\left\{\begin{array}{lcl}{S}_{0} & + & [{\rm{\cos }}(2\alpha ){{\rm{\cos }}}^{2}(2\theta )+{\rm{\sin }}(2\alpha ){\rm{\cos }}(2\theta ){\rm{\sin }}(2\theta )]\\ {S}_{1} & + & [{\rm{\cos }}(2\alpha ){\rm{\cos }}(2\theta ){\rm{\sin }}(2\theta )+{\rm{\sin }}(2\alpha ){{\rm{\sin }}}^{2}(2\theta )]\\ {S}_{2} & + & [{\rm{\sin }}(2\alpha ){\rm{\cos }}(2\theta )-{\rm{\cos }}(2\alpha ){\rm{\sin }}(2\theta )]{S}_{3}\end{array}\right\}.$$

As described in the image capture, the transmission axis of LP is fixed at 45°, and the fast axis of QWP is rotated successively to 0°, 22.5°, 45°, 67.5° and 90°. By substituting the designed QWP and LP angles into Eq. (5), the intensity information detected by CMOS can be expressed as

6 $$\left.\begin{array}{l}{I}_{{0}^{\circ },4{5}^{\circ }}={S}_{0}/2+{S}_{3}/2\\ {I}_{22.{5}^{\circ },4{5}^{\circ }}={S}_{0}/2+{S}_{1}/4+{S}_{2}/4+\sqrt{2}{S}_{3}/4\\ {I}_{4{5}^{\circ },4{5}^{\circ }}={S}_{0}/2+{S}_{2}/2\\ {I}_{67.{5}^{\circ },4{5}^{\circ }}={S}_{0}/2-{S}_{1}/4+{S}_{2}/4-\sqrt{2}{S}_{3}/4\\ {I}_{9{0}^{\circ },4{5}^{\circ }}={S}_{0}/2-{S}_{3}/2\end{array}\right\}.$$

Then, the four Stokes parameters of the object reflected light can be solved through

7 $$\left.\begin{array}{l}{S}_{0}={I}_{{0}^{\circ },4{5}^{\circ }}+{I}_{9{0}^{\circ },4{5}^{\circ }}\\ {S}_{1}=2\left({I}_{22.{5}^{\circ },4{5}^{\circ }}-{I}_{67.{5}^{\circ },4{5}^{\circ }}\right)-\sqrt{2}\left({I}_{{0}^{\circ },4{5}^{\circ }}-{I}_{9{0}^{\circ },4{5}^{\circ }}\right)\\ {S}_{2}=2{I}_{4{5}^{\circ },4{5}^{\circ }}-\left({I}_{{0}^{\circ },4{5}^{\circ }}+{I}_{9{0}^{\circ },4{5}^{\circ }}\right)\\ {S}_{3}={I}_{{0}^{\circ },4{5}^{\circ }}-{I}_{9{0}^{\circ },4{5}^{\circ }}\end{array}\right\}.$$

Herein, we further calculate five other well-known polarization parameters, including the angle of linear polarization (*AoLP*), the angle of circular polarization (*AoCP*), the degree of linear polarization (*DoLP*), the degree of circular polarization (*DoCP*) and the degree of polarization (*DoP*). The five polarization parameters are calculated as follows:

8 $$\left.\begin{array}{l}AoLP={\rm{\arctan }}\left({S}_{2}/{S}_{1}\right)/2\\ AoCP={\rm{\arcsin }}\left({S}_{3}/{S}_{0}\right)/2\\ DoLP=\sqrt{{S}_{1}^{2}+{S}_{2}^{2}}/{S}_{0}\\ DoCP=\left|{S}_{3}\right|/{S}_{0}\\ DoP=\sqrt{{S}_{1}^{2}+{S}_{2}^{2}+{S}_{3}^{2}}/{S}_{0}\end{array}\right\}.$$

**Fig. 6:**
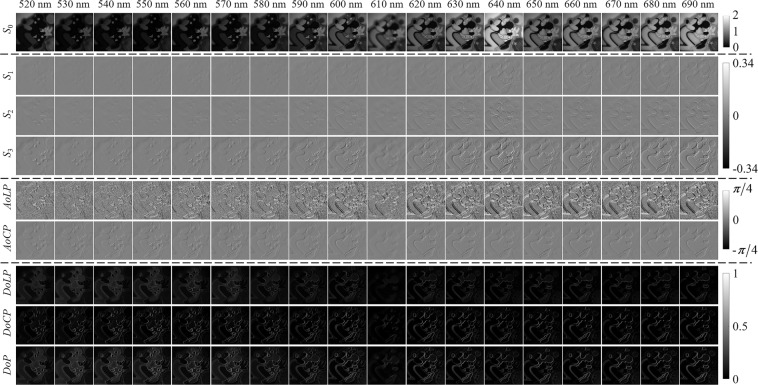
The images of four Stokes and five polarization parameters calculated for an object at 18 spectral bands.

**Fig. 7:**
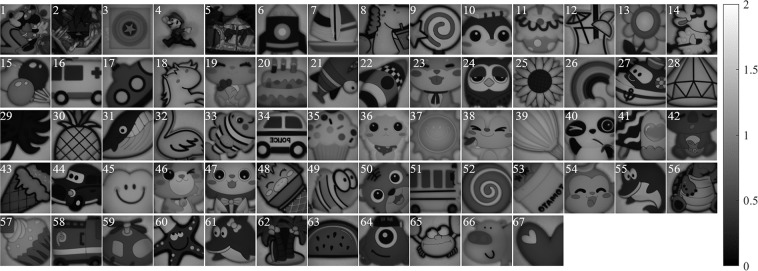
The *S*_0_ images of all 67 objects with different appearance at the spectral band of 600 nm.

Figure 6 shows the images of four Stokes parameters *S*_0_, *S*_1_, *S*_2_, *S*_3_ and five polarization parameters *AoLP*, *AoCP*, *DoLP*, *DoCP*, *DoP* calculated for an object at 18 spectral bands. Combining the theoretical values of parameters with the actual values of images, the *S*_0_ images are displayed in the grayscale range of 0 to 2. The *S*_1_, *S*_2_ and *S*_3_ images are displayed in the grayscale range of −0.34 to 0.34. The *AoLP* and *AoCP* images are displayed in the grayscale range of −*π*/4 to *π*/4. The *DoLP*, *DoCP* and *DoP* images are displayed in the grayscale range of 0 to 1. It is found that *S*_0_ can reflect the overall appearance of the object, and other parameters can reflect the surface texture of the object. Selecting the spectral band of 600 nm, the *S*_0_ images of all 67 objects are shown in Fig. 7 to reflect their different appearance.

## Data Records

The raw experimental images of all 67 objects are packaged into “FSPMI experimental data.zip” and deposited to the *figshare*^28^. The folder labeled as “Object_xx” contains images captured for each object. For example, the folder named “Object_01” contains images captured for the first object. The subfolders and image files are described in Table 2. A total of 36582 images are captured for 67 objects, accounting for 571 GB.

**Table 2:** The raw experimental images in each folder labeled as “Object_xx”.

Folder or file name	Description
black	Contain images with the light source off
520/530/…/690	Contain images under a bandpass filter with the central wavelength of 520/530/…/690 nm
QWP_0_LP_45	Contain images at 0° QWP fast axis and 45° LP transmission axis
QWP_22.5_LP_45	Contain images at 22.5° QWP fast axis and 45° LP transmission axis
QWP_45_LP_45	Contain images at 45° QWP fast axis and 45° LP transmission axis
QWP_67.5_LP_45	Contain images at 67.5° QWP fast axis and 45° LP transmission axis
QWP_90_LP_45	Contain images at 90° QWP fast axis and 45° LP transmission axis
1/2/3/4/5/6.bmp	Captured image on CMOS detector

The processed image data of all 67 objects is packaged into “FSPMI dataset.zip” and also deposited to the *figshare*^28^. The image data for each object is stored separately in a file labeled as “Object_xx.mat”. For example, the file labeled as “Object_01.mat” stores image data for the first object. Table 3 lists the variable names, sizes and corresponding descriptions of the data in each object file. The data in each file includes a set of spectral bands, five sets of preprocessed images, four sets of Stokes images, two sets of polarization angle images and three sets of polarization degree images.

**Table 3:** The processed image data in each file labeled as “Object_xx.mat”.

Variable name	Size	Description
band	1 × 18	The central wavelengths of the 18 spectral bands
AoCP	512 × 512 × 18	The angle of circular polarization
AoLP	512 × 512 × 18	The angle of linear polarization
DoCP	512 × 512 × 18	The degree of circular polarization
DoLP	512 × 512 × 18	The degree of linear polarization
DoP	512 × 512 × 18	The degree of polarization
QWP_0_LP_45	512 × 512 × 18	Preprocessed images at 0° QWP fast axis and 45° LP transmission axis
QWP_22p5_LP_45	512 × 512 × 18	Preprocessed images at 22.5° QWP fast axis and 45° LP transmission axis
QWP_45_LP_45	512 × 512 × 18	Preprocessed images at 45° QWP fast axis and 45° LP transmission axis
QWP_67p5_LP_45	512 × 512 × 18	Preprocessed images at 67.5° QWP fast axis and 45° LP transmission axis
QWP_90_LP_45	512 × 512 × 18	Preprocessed images at 90° QWP fast axis and 45° LP transmission axis
S0	512 × 512 × 18	The first Stokes parameter *S*_0_
S1	512 × 512 × 18	The second Stokes parameter *S*_1_
S2	512 × 512 × 18	The third Stokes parameter *S*_2_
S3	512 × 512 × 18	The fourth Stokes parameter *S*_3_

## Technical Validation

The FSPMI dataset is intercepted by 400×400 spatial pixels and further applied to validate several existing compressive full-Stokes PMI methods^17–19^ to demonstrate data reliability. These compressive full-Stokes PMI methods differ in terms of experimental systems and reconstruction methods. To modulate and compress the four Stokes parameters, the compressive full-Stokes polarization and flexible multispectral four-dimensional imaging (CFPMI) method^17^ employs a QWP, an LP and an LCTF. Furthermore, the CFPMI method accurately solves *S*_0_ and then reconstructs *S*_1_, *S*_2_, *S*_3_ based on CS theory^14^. The four-dimensional compressed spectropolarimetric imaging (FDCSPI) method^18^ adopts a QWP and an LCTF to modulate and compress the four Stokes parameters. Moreover, the FDCSPI method firstly reconstructs *S*_1_, *S*_2_, *S*_3_ based on CS theory and then solves *S*_0_. The full polarization-compressed multispectral imaging (FPCMI) method^19^ also modulates and compresses the four Stokes parameters by a QWP and an LCTF. Nevertheless, the FPCMI method first reconstructs *S*_0_ and then *S*_1_, *S*_2_, *S*_3_ based on feature scaling and CS theory.

The above methods are validated for all 67 objects in the FSPMI dataset. For each object data, three polarization modulation is performed in the CFPMI, FDCSPI and FPCMI methods. In the CFPMI method, the transmission axis of LP is set to 45°, 135° and 135° respectively, while the fast axis of QWP is randomly set to the same angle in the first two modulation and is changed in the third modulation. In the FDCSPI and FPCMI methods, three angles are randomly set for the fast axis of QWP. In a word, the fast axis angle of QWP is randomly set for both the three methods and 67 object data. Similarly, the transmission axis of linear polarization of the LCTF incidence plane is set to 0° in the above three methods. Meanwhile, all spectral bands of interest are provided for each object by freely switching the central wavelength of the LCTF. In addition, all the reconstruction based on CS theory use the discrete W transform basis^29^ and the two-step iterative shrinkage/thresholding (TwIST) algorithm^30^ with iteration accuracy of 0.005.

**Fig. 8:**
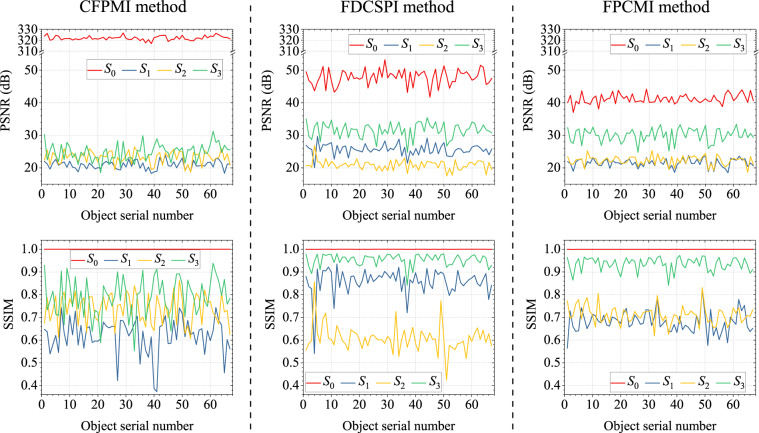
The averaged PSNR and SSIM values of the four Stokes parameters for all 67 objects in validating the CFPMI, FDCSPI and FPCMI methods.

**Fig. 9:**
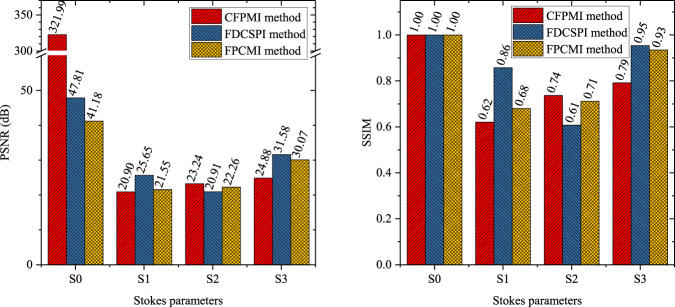
The averaged PSNR and SSIM values of each Stokes parameter for the CFPMI, FDCSPI and FPCMI methods.

Figure 8 shows the reconstruction results of the four Stokes parameters for all 67 objects in validating the CFPMI, FDCSPI and FPCMI methods. The reconstruction results are reflected by the peak signal to noise ratio (PSNR) and structural similarity (SSIM) values averaged over 18 spectral bands. For better comparison, the PSNR and SSIM values of each Stokes parameter are averaged over 67 objects. The averaged PSNR and SSIM values of each Stokes parameter for the CFPMI, FDCSPI and FPCMI methods are shown in Fig. 9. The averaged PSNR values are all greater than 20 dB, and the averaged SSIM values are all greater than 0.6. Therefore, the reliability of FSPMI dataset is effectively validated in the CFPMI, FDCSPI and FPCMI methods.

To improve the reconstruction results, the polarization modulation strategy can be further designed based on CS theory. In addition, the diversity of the 67 objects provides flexibility for studying compressive full-Stokes PMI methods in various scenarios. Meanwhile, the richness of the 67 objects contributes to the development of machine learning-based reconstruction methods in compressive full-Stokes PMI.

## Usage Notes

The FSPMI dataset can serve as source data for both compressive polarization imaging and compressive multispectral imaging. This dataset significantly settles the problem of inconsistency and time-consuming in data acquisition, including simulation data and experimental data. Simulation and experiment are usually combined to investigate the effectiveness of imaging methods in terms of encoding strategies and reconstruction algorithms. In general, the encoding method is different in simulation and experiment. The numerical calculation performs the simulation encoding, while the imaging system performs the experimental encoding.

Therefore, in polarization imaging, the captured polarization encoded images and the derived full-Stokes parameter images can be involved in different requirements. Less captured polarization encoded images can be used as experimental data to reconstruct full-Stokes parameter images. The derived full-Stokes parameter images can be used as simulation data to numerically calculate less polarization encoded images, and then to reconstruct full-Stokes parameter images. Furthermore, the reconstructed and derived full-Stokes parameter images are compared from various aspects to evaluate the imaging method. Meanwhile, the polarization angle images and polarization degree images can provide additional references for the evaluation.

Multispectral imaging mainly involves captured and derived light intensity images at 18 spectral bands. The central wavelength of 18 spectral bands ranges from 520 nm to 690 nm with 10 nm intervals and 10 nm bandwidths. Thus, the images in each spectral band are regarded as ideal narrowband images. Moreover, multispectral images can be used as simulation data to numerically calculate less spectral encoded images, and then to reconstruct multispectral images. Similarly, the reconstructed and unencoded multispectral images are compared sufficiently to evaluate the imaging method. Notably, image registration may be required to eliminate the slight drift of object images in different spectral bands caused by the position error of multiple filters.

In addition, the diversity of 67 objects ensures that the FSPMI dataset is suitable for developing machine learning-based imaging methods. The 67 objects are plastic cartoon images, including persons, animals, food, vehicles and other things. According to the object’s structural complexity, researchers can test and select appropriate objects to meet the requirements of the imaging method.

## Data Availability

The experimental data are processed using MATLAB R2019b software to generate the FSPMI dataset. The software code and supporting files that generate the dataset are packaged into “FSPMI generation code.zip” and deposited to the *figshare*^28^. The file labeled as “FSPMI_generation_main.m” is the main program to process the experimental data to further generate the dataset. The file labeled as “text2trans.m” is the function required to run the main program, implementing the interpolation of the various spectral responses to the same wavelengths. Spectral response files (.txt) for all devices named by their items are provided in the folder “Optical devices”, which is also called when running the main program. In addition, the toolboxes named “curvefit”, “eml” and “matlab” are necessary to run the main program.

## References

[1] Islam MN, Tahtali M, Pickering M (2021). Specular reflection detection and inpainting in transparent object through MSPLFI. Remote. Sens..

[2] Islam MN, Tahtali M, Pickering M (2020). Hybrid fusion-based background segmentation in multispectral polarimetric imagery. Remote. Sens..

[3] Zhang J, Tan J, Zhang Y (2017). Joint sparse tensor representation for the target detection of polarized hyperspectral images. IEEE Geosci. Remote. Sens. Lett..

[4] Yao H (2022). Combination of hyperspectral and quad-polarization SAR images to classifymarsh vegetation using stacking ensemble learning algorithm. Remote. Sens..

[5] Tu C (2021). Synergetic classification of coastal wetlands over the Yellow River Delta with GF-3 full-polarization SAR and Zhuhai-1 OHS hyperspectral remote sensing. Remote. Sens..

[6] Liu M, Sun Z, Lu S, Omasa K (2022). Combining multiangular, polarimetric, and hyperspectral measurements to estimate leaf nitrogen concentration from different plant species. IEEE Transactions on Geosci. Remote. Sens..

[7] Dremin V (2021). Skin complications of diabetes mellitus revealed by polarized hyperspectral imaging and machine learning. IEEE Transactions on Med. Imaging.

[8] He C (2015). Characterizing microstructures of cancerous tissues using multispectral transformed Mueller matrix polarization parameters. Biomed. Opt. Express.

[9] Quan N, Zhang C, Mu T, Li S, You C (2021). Snapshot spectroscopic Mueller matrix polarimetry based on spectral modulation with increased channel bandwidth. Opt. Express.

[10] Lv X (2021). Channeled imaging spectropolarimeter reconstruction by neural networks. Opt. Express.

[11] Mu T (2019). Optimized design, calibration, and validation of an achromatic snapshot full-Stokes imaging polarimeter. Opt. Express.

[12] Mu T (2022). Snapshot hyperspectral imaging polarimetry with full spectropolarimetric resolution. Opt. Lasers Eng..

[13] Chen Z (2019). Coded aperture snapshot linear-Stokes imaging spectropolarimeter. Opt. Commun..

[14] Baraniuk RG (2007). Compressive sensing [lecture notes]. IEEE Signal Process. Mag..

[15] Ren W, Fu C, Wu D, Xie Y, Arce GR (2019). Channeled compressive imaging spectropolarimeter. Opt. Express.

[16] Xu Z (2022). Snapshot compressive imaging full-Stokes polarimeter. Opt. Commun..

[17] Fan A (2023). Compressive full-Stokes polarization and flexible hyperspectral imaging with efficient reconstruction. Opt. Lasers Eng..

[18] Fan A (2022). Four-dimensional compressed spectropolarimetric imaging. Signal Process..

[19] Fan A, Xu T, Wang X, Xu C, Zhang Y (2020). Scaling-based two-step reconstruction in full polarization-compressed hyperspectral imaging. Sensors.

[20] Rubin NA (2019). Matrix Fourier optics enables a compact full-Stokes polarization camera. Science.

[21] Zhang C (2021). High efficiency all-dielectric pixelated metasurface for near-infrared full-Stokes polarization detection. Photonics Res..

[22] Song Q (2021). Broadband decoupling of intensity and polarization with vectorial Fourier metasurfaces. Nat. Commun..

[23] Kim JH, Stolte M, Würthner F (2022). Wavelength and polarization sensitive synaptic phototransistor based on organic n-type semiconductor/supramolecular J-aggregate heterostructure. ACS Nano.

[24] Altaqui A (2021). Mantis shrimp-inspired organic photodetector for simultaneous hyperspectral and polarimetric imaging. Sci. Adv..

[25] Qin G, Zhang P, Sun M, Fu W, Cai C (2022). Comprehensive spectral libraries for various rabbit eye tissue proteomes. Sci. Data.

[26] Hristu R (2022). PSHG-TISS: A collection of polarization-resolved second harmonic generation microscopy images of fixed tissues. Sci. Data.

[27] Arad, B. & Ben-Shahar, O. Sparse recovery of hyperspectral signal from natural RGB images. In *European Conference on Computer Vision*, 19–34, 10.1007/978-3-319-46478-7_2 (Springer, 2016).

[28] Fan A (2023). figshare.

[29] Bi G, Zeng Y, Lin Z (2003). Odd-factor algorithms for multidimensional discrete W transform. Circuits, Syst. Signal Process..

[30] Bioucas-Dias JM, Figueiredo MAT (2007). A new TwIST: Two-step iterative shrinkage/thresholding algorithms for image restoration. IEEE Transactions on Image Process..

